# Tensile deformation behavior of twist grain boundaries in CoCrFeMnNi high entropy alloy bicrystals

**DOI:** 10.1038/s41598-020-77487-z

**Published:** 2021-01-11

**Authors:** Hyunsoo Lee, Mitra Shabani, Garrett J. Pataky, Fadi Abdeljawad

**Affiliations:** 1 Department of Mechanical Engineering, Clemson University, Clemson, SC 29634 USA; 2 Department of Materials Science and Engineering, Clemson University, Clemson, SC 29634 USA

**Keywords:** Metals and alloys, Surfaces, interfaces and thin films

## Abstract

High entropy alloys (HEA) are a class of materials that consist of multiple elemental species in similar concentrations. The use of elements in far from dilute concentrations introduces a multi-dimensional composition design space by which the properties of metallic systems can be tailored. While the mechanical behavior of HEAs has been the subject of active research recently, the role of grain boundaries (GBs) in their deformation behavior remains poorly understood. Motivated by recent experiments on HEAs demonstrating that GBs act as nucleation sites for deformation twins, herein, we leverage atomistic simulations to construct a series of equiatomic CoCrFeMnNi HEA bicrystals with $\langle 110 \rangle$ and $\langle 111 \rangle$ symmetric twist GBs and examine their tensile behavior and underlying deformation mechanisms at 77 K. Simulation results reveal that plastic deformation proceeds by the nucleation of partial dislocations from GBs, which then grow with further loading by bowing into the bulk crystals leaving behind stacking faults. Variations in the nucleation stress exist as function of GB character, defined in this work by the twist angle. Our results provide future avenues to explore GBs as a microstructure design tool to develop HEAs with tailored properties.

## Introduction

Unlike conventional metallic alloys, which are comprised of a base (solvent) and alloying (solute) elements, high entropy alloys (HEAs) are a class of structural alloys first introduced by Cantor et al.^1^ and Yeh et al.^2^ that consist of multiple principal elements in equiatomic or near equiatomic concentrations^3–5^. Owing to their multi-principal element compositions, HEAs are characterized by unique combinations of properties, including mechanical strength^2,6,7^, hardness^8,9^, and wear^10,11^ and corrosion resistance^12–15^. The use of multiple principal elements in far-from dilute concentrations unlocks infinite possibilities of combinations of properties and functionalities that are not typically found in conventional metallic alloys.

Numerous recent studies on 3d transition metal HEAs, such as CoCrFeMnNi^1,16–18^, CoCrFeNi^16,19^, CoFeMnNi^16^, CoCrMnNi^16^, and $\hbox {Co}_{0.25}$Cr$_{0.1}$Fe$_{2}$Mn$_{1.35}$Ni$_{1.3}$^20^, have explored the impact of temperature^21,22^, grain size^23–25^, alloy composition^26–28^, strain rate^29^, strain levels^30^, and phase transformations^31,32^ on the mechanical behavior of these alloys. The equiatomic CoCrFeMnNi, or so-called Cantor, alloy is a face-centered cubic (FCC) single-phase alloy that has been regarded as a model system to many single-phase HEAs. The ability to decipher the fundamental mechanisms controlling the unique properties of the Cantor alloy would improve our understanding of this new class of metallic alloys, thus enabling their use in many engineering applications^5,33^.

Understanding the mechanical properties of the Cantor alloy has been the subject of active research efforts. Its elastic constants, determined using experimental^33,34^ and computational^35^ techniques, have been found to exhibit similar elastic anisotropy as Fe^36^. Further, the Cantor alloy was found to exhibit simultaneous mechanical strength and ductility^17,22,23,37–44^ with yield strength values that are comparable to Ni-based superalloys and steels, but lower than aluminum-containing and refractory metal-based HEAs^21,36^. Gali and George^45^ have shown that the yield and ultimate strengths and ductility all increase simultaneously with decreasing temperature down to 77 K. The lack of tradeoff between strength and ductility and the exceptional fracture-toughness of the Cantor alloy at low temperatures have been attributed to deformation induced nanotwinning as an additional mode of plastic deformation^17,21,22,46^.

Extensive deformation-induced twinning has been observed in the Cantor alloy, as well as other FCC HEAs, at various temperatures, including 77 K, and with grain sizes ranging from millimeter down to sub-micrometer^17,21,47,48^. Such mechanical twins have been observed to be nanometric in thickness and inter-twin spacings^17,38,47^. Recently, experimental techniques employing diffraction-contrast scanning-transmission electron microscopy have been employed to measure dissociation distance of $\langle 1 1 0 \rangle$ dislocations in HEAs^49^. The extensive twinning of the Cantor alloy at low temperatures has been attributed to its low stacking fault energy (SFE)^21^, which was determined to be in the range of 20–25 $\hbox {mJ m}^{-2}$^50,51^. Laplanche et al.^42^ experimentally determined the stress level at which mechanical twins are observed in the Cantor alloy ($\approx 720$ MPa), leading to an estimated critical resolved shear stress of $235\pm 10$ MPa. Further, they tracked the evolution of twin widths, mean twin spacing, and twin volume fraction as a function of applied strain.

**Figure 1:**
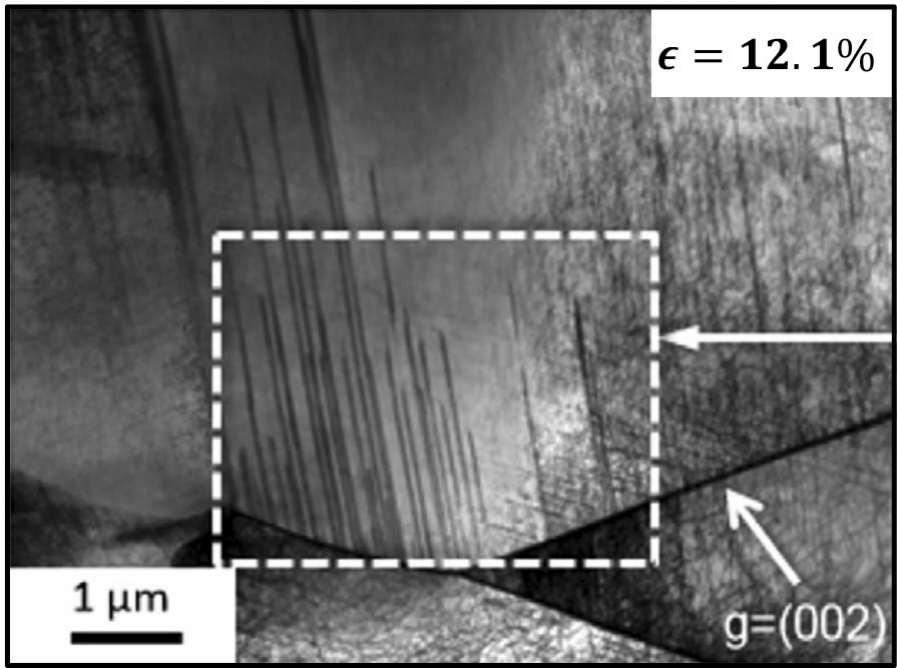
A bright-field TEM image depicting nanotwin nucleation from a GB in a CoCrFeMnNi HEA at a true tensile strain $\epsilon = 12.1\%$ and temperature of 77 K. Taken from Ref.^42^ with permission from Elsevier.

While the aforementioned studies shed light on the mechanical behavior of HEAs, the role of materials defects in general and grain boundaries (GBs) in specific in deformation mechanisms remains poorly understood. The experiments by Bell and Cahn^52,53^ on Zn crystals highlighted the impact of defects on twin nucleation by observing that carefully prepared Zn single crystals can be mechanically loaded to much higher levels than those at which twins typically form in crystals with defects. Nucleation of mechanical twins from GBs have been observed in a wide range of metallic systems including Cu^54^, Mg-based^55,56^, and Ti-based^57,58^ systems. Very recently, experimental studies on HEAs have revealed nanotwin nucleation from GBs^38,42,59^. For example, Fig. 1 shows a bright-field transmission electron microscope (TEM) image from a study by Laplanche et al.^42^ depicting the nucleation of deformation nanotwins from a GB in the Cantor alloy at a true tensile strain $\epsilon = 12.1\%$ and temperature of 77 K.

The primary goal of this study is to explore the role of GBs in the deformation mechanisms of the equiatomic CoCrFeMnNi HEA. To this end, we leveraged molecular dynamics (MD) to simulate the tensile deformation behavior of a series of Cantor alloy bicrystals with $\langle 110 \rangle$ and $\langle 111 \rangle$ symmetric twist GBs at 77 K. Twist GBs were chosen in this study as their geometric construction allows us to investigate the behavior of several GB types in bicrystal systems while maintaining a common crystal direction along the loading axis for all these systems.

## Methods

A series of Cantor alloy bicrystals with $\langle 110 \rangle$ and $\langle 111 \rangle$ symmetric twist GBs (STGBs) were generated and studied using a second nearest-neighbor modified embedded atom method interatomic potential fit to the energetics of all unary, binary, and ternary combinations of the CoCrFeMnNi system^26^. This potential reproduces experimental mechanical properties and solid solution hardening effect in non-equiatomic CoCrFeMnNi HEAs at arbitrary compositions. All MD simulations reported in this work were performed using LAMMPS atomistic simulation package^60^ and visualizations of atomistic structures were generated using OVITO^61^. Analysis of dislocation structures was performed using the dislocation extraction algorithm (DXA)^62^. The OVITO implementations of the centrosymmetry parameter, common neighbor analysis (CNA), and polyhedral template matching (PTM) algorithm^63^ were used to identify crystal structures and ordering in the atomistic systems, i.e., FCC, hexagonal close packing (HCP).

**Figure 2:**
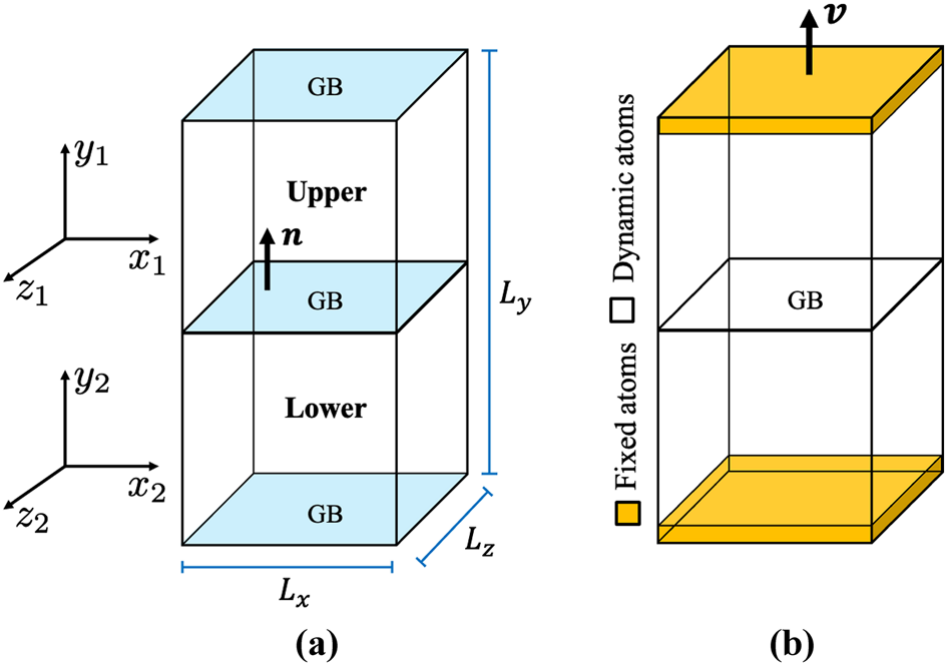
A schematic depicting **(a)** the geometry of the atomistic bicrystal system and **(b)** the configuration used in the mechanical loading simulations.

The first step in our approach was the construction of Cantor alloy bicrystals with prescribed GB geometries. For each bicrystal, a fully periodic Cantor alloy atomistic system was created from two half crystals, each of which was rotated such that the resulting planar GB between the halves had the specified misorientation angle. Figure 2a shows a schematic representation of the bicrystal geometry used in this work, where the GB plane normal $\mathbf{{n}}$ aligns with the *y*-axis of both crystals, Table 1 lists the *x*-axis for the upper ($x_1$) and lower ($x_2$) crystals for each of the [110] and [111] STGBs examined in this work. A sequence of relative displacements between the upper and lower half-crystals was used in conjunction with atom deletions and conjugate gradient energy minimizations to identify a low energy GB configuration. The systems were allowed to expand or contract in the perpendicular direction to the GB plane. In order to capture the periodicity of a GB atomic structure, the target dimensions of the bicrystal geometry were set to $L_x = L_z = 100$ Å and $L_y = 600$ Å. However, the dimension of each sample varied so as to accommodate an integer number of unit cells necessary to model each specific GB. The number of atoms in each sample ranged from 600,000 to 1 million. After the bicrystal construction step, we thermally equilibrated the systems by heating the simulation box to a temperature of 77 K and holding for 2 ns with a target pressure of zero, allowing the system to expand or contract as appropriate. This was achieved by performing isothermal and isobaric integration with a 1 fs time step to march atoms’ positions and velocities using a Nosé-Hoover thermostat and barostat^64^. This creates a trajectory in phase space that is consistent with the isothermal-isobaric (NPT) ensemble. To damp undesirable oscillations in the system’s temperature and pressure, the drag option in LAMMPS was used.

**Table 1:** For the bicrystal geometry depicted in Fig. 2a, the crystal *x*-axis for the upper $x_1$ and lower $x_2$ crystals. The crystal *z*-axis is obtained using ${\mathbf{z}} = {\mathbf{x}} \times {\mathbf{y}}$, where the GB plane normal is along the crystal *y*-axis. The *x*-axis for single crystal systems with a [110] ($\hbox {SC}_{110}$) and [111] ($\hbox {SC}_{111}$) loading axis is also listed.

[110] STGBs			[111] STGBs		
$\Sigma$	${\mathbf{x}}_1$ / ${\mathbf{x}}_2$	$\theta$	$\Sigma$	${\mathbf{x}}_1$ / ${\mathbf{x}}_2$	$\theta$
value	[hkl]$_{\text {upper}}$ / [hkl]$_{\text {lower}}$	$(^{\circ })$	value	[hkl]$_{\text {upper}}$ / [hkl]$_{\text {lower}}$	$(^{\circ })$
3	[1 $\overline{1}$ $\overline{1}$] / [$\overline{1}$ 1 $\overline{1}$]	70.53	3	[1 1 $\overline{2}$] / [$\overline{1}$ $\overline{1}$ 2]	60.00
9	[2 $\overline{2}$ $\overline{1}$] / [$\overline{2}$ 2 $\overline{1}$]	38.95	7	[1 4 $\overline{5}$] / [4 1 $\overline{5}$]	38.21
17	[2 $\overline{2}$ $\overline{3}$] / [$\overline{2}$ 2 $\overline{3}$]	86.60	13	[2 5 $\overline{7}$] / [5 2 $\overline{7}$]	27.80
19	[3 $\overline{3}$ $\overline{1}$] / [$\overline{3}$ 3 $\overline{1}$]	26.53	19	[1 7 $\overline{8}$] / [7 1 $\overline{8}$]	46.83
27	[1 $\overline{1}$ $\overline{5}$] / [$\overline{1}$ 1 $\overline{5}$]	31.60	21	[1 4 $\overline{5}$] / [$\overline{1}$ 5 $\overline{4}$]	21.79
33	[2 $\overline{2}$ $\overline{5}$] / [$\overline{2}$ 2 $\overline{5}$]	58.98	31	[4 7 $\overline{11}$] / [7 4 $\overline{11}$]	17.90
41	[4 $\overline{4}$ $\overline{3}$] / [$\overline{4}$ 4 $\overline{3}$]	55.88	39	[2 5 $\overline{7}$] / [$\overline{2}$ 7 $\overline{5}$]	32.20
43	[3 $\overline{3}$ $\overline{5}$] / [$\overline{3}$ 3 $\overline{5}$]	80.63	57	[1 7 $\overline{8}$] / [$\overline{1}$ 8 $\overline{7}$]	13.17
51	[5 $\overline{5}$ $\overline{1}$] / [$\overline{5}$ 5 $\overline{1}$]	16.10	93	[4 7 $\overline{11}$] / [$\overline{4}$ 11 $\overline{7}$]	42.10
201	[10 $\overline{10}$ $\overline{1}$] / [10 $\overline{10}$ 1]	8.10	111	[1 10 $\overline{11}$] / [$\overline{1}$ 11 $\overline{10}$]	9.43
$\hbox {SC}_{{110}}$	**x** = [2 $\overline{2}$ $\overline{5}$]	–	$\hbox {SC}_{{111}}$	**x** = [1 1 $\overline{2}$]	–

After the initial construction and thermal equilibration steps, uniaxial tension up to 12% was performed along the *y*-axis of each sample at 77 K, while the normal pressure was set to zero in the transverse directions using the NPT ensemble, thus allowing for lateral expansion/shrinkage. This was achieved by defining two slabs in which the atoms were fixed in their positions relative to each another, refer to Fig. 2b for a schematic representation of the mechanical deformation simulations. The slabs used to impose boundary conditions and forces on the dynamic atoms had a thickness of $\approx 25$ Å. Uniaxial tension was applied by moving all fixed atoms of the upper slab with the same constant velocity of $v = 0.3$ Å/ps along the *y*-direction, while the lower slab remained fixed (see Supplementary Fig. S1 online for a study of strain rate effects). The nominal strain along the *y*-direction of the sample was calculated as $\epsilon _{yy} = \Delta L_y /L_y(0)$, where $L_y(0)$ is the initial length, and the stress tensor averaged over all dynamic atoms was computed using the standard virial expression.

## Results

**Figure 3:**
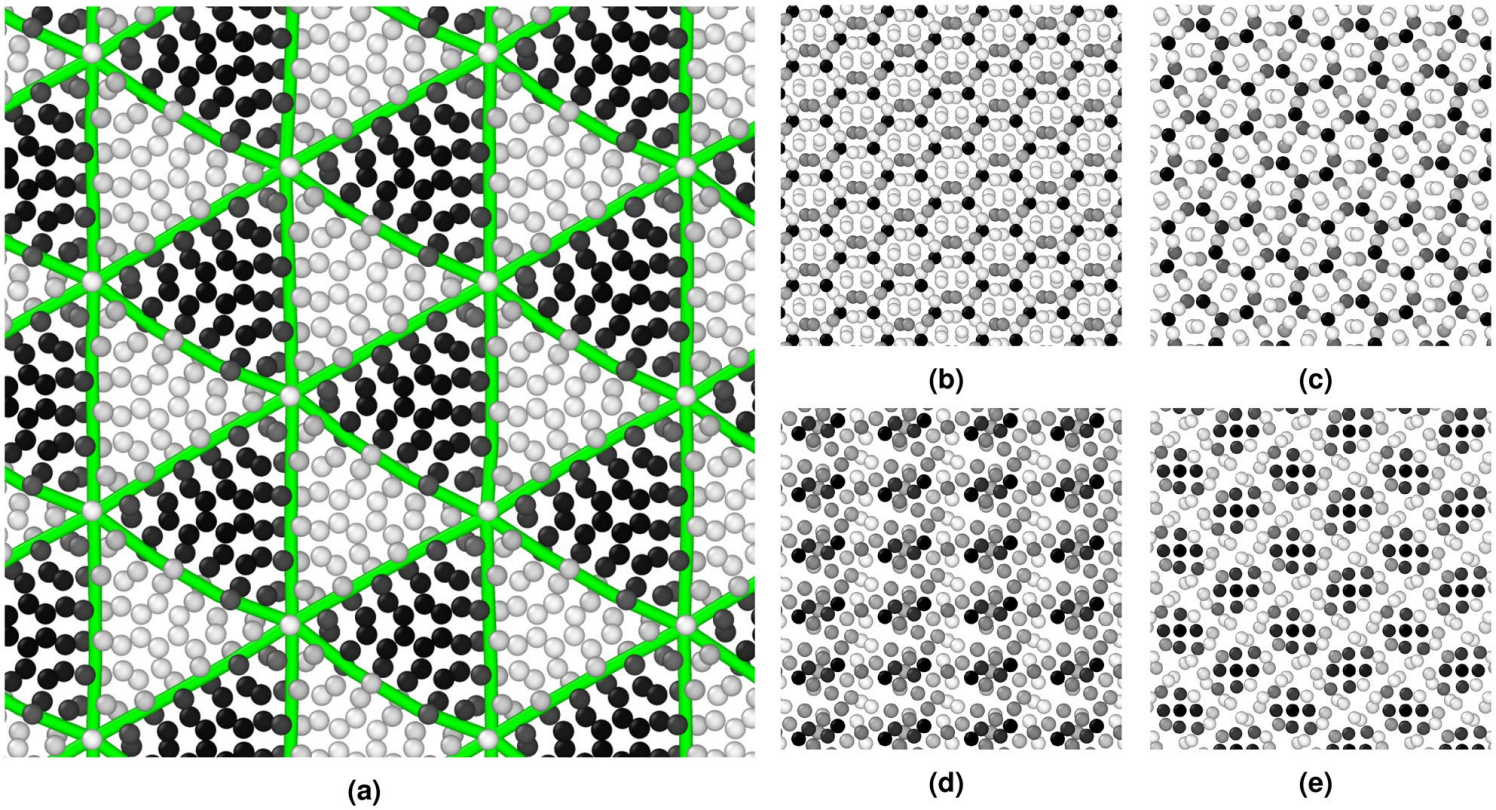
View of the GB plane showing the structure for select **(a)–(c)** [111] and **(d)–(e)** [110] STGBs. The structure of **(a)** $\Sigma 111$, **(b)** $\Sigma 7$, **(c)** $\Sigma 39$, **(d)** $\Sigma 9$, and **(e)** $\Sigma 17$ STGBs are shown. Atoms are colored according to the centrosymmetry parameter, where lighter colors indicate greater deviation from local FCC ordering. For the $\Sigma 111$ [111] STGB in **(a)**, interfacial dislocation network (green) extracted using DXA are shown.

### Twist grain boundary structures

The GBs chosen for this study were $\langle 110 \rangle$ and $\langle 111 \rangle$ STGBs with a wide range of twist angles and, as a result, GB structures. Table 1 lists the ten $\langle 110 \rangle$ and ten $\langle 111 \rangle$ GBs explored in this work in the order of increasing $\Sigma$ value, where $1/\Sigma$ represents the percent of lattice points that are in coincidence (i.e., the coincidence site lattice description)^65^. Each of the two half crystals was rotated about the twist axis (i.e., the crystal *y*-axis given by [110] or [111]) by an equal and opposite angle $\theta /2$, where $\theta$ defines the total twist angle. The *x*-direction for each of the crystals after the rotation is also shown in Table 1. Projected views of the GB plane depicting the 0K equilibrated structures before mechanical deformation for five selected GBs are shown in Fig. 3. Atoms are colored according to the centrosymmetry parameter, where lighter atoms indicate greater deviations from local FCC ordering (see Supplementary Fig. S2 online for the same figure, but with atoms colored according to element type). The atomic structures for the $\Sigma 111$, $\Sigma 7$, and $\Sigma 39$ [111] GBs are shown in Fig. 3a–c, respectively, whereas Fig. 3d–e shows respectively $\Sigma 9$ and $\Sigma 17$ [110] GBs. In Fig. 3a representing the structure of the $\Sigma 111$ [111] STGB, DXA was used to reveal and characterize the interfacial dislocation network, shown in green lines, within the GB plane. For this low-twist angle GB, the boundary is comprised of a grid of 1/6 $\langle 112 \rangle$ partial screw dislocations with alternating regions of FCC (atoms colored in black) and HCP (atoms colored in gray) structures. Similar interfacial dislocation networks in low-twist angle GBs have been recently observed in atomistic calculations^66^. Chen et al.^67^ examined the GB character distribution in the Cantor alloy during recrystallization and showed a high density of twin $\Sigma 3$ and low-angle GBs. In a recent study by Lin et al.^68^, high-resolution microscopy was employed to study boundary dislocations at low-angle ($\sim 6^{\circ }$) GBs in the Cantor alloy and determine their Burgers vectors and dislocation spacing.

Here, it is worth noting that while the GB generation algorithm used in this work resulted in equilibrated GB structures at 0K by performing a series of rigid body translations within the GB plane, i.e., so-called $\gamma$-surface method^69,70^, chemical equilibration was not performed. Such a step includes probing the preferential sites with the GB plane for each of the elemental species, sampling and optimizing the GB atomic density, and probing segregation effects of one or more elemental species to the GB. The phase space of possible chemical configurations for the equiatomic five-component Cantor system is large and beyond the scope of this work. As a result, the GB structures explored in this work can be regarded as metastable ones. Indeed, the multiplicity of metastable GB states has been the subject of active research recently for its influence on the behavior and properties of metallic systems^71,72^. Very recently, Frolov et al.^73^ and Zhu et al.^74^ developed an evolutionary algorithm that performs a grand-canonical GB structure search and were able to identify a plethora of GB phases with different structures in *pure* metals. Also, Wynblatt and Chatain^75^ in a recent computational study showed using Monte Carlo, molecular dynamics, and lattice statics methods that both Cr and Mn segregate to GBs in the CoCrFeMnNi Cantor alloy, and such segregation leads to depletion of bulk compositions when the grain size of these alloys is reduced into the nanoscale.

**Figure 4:**
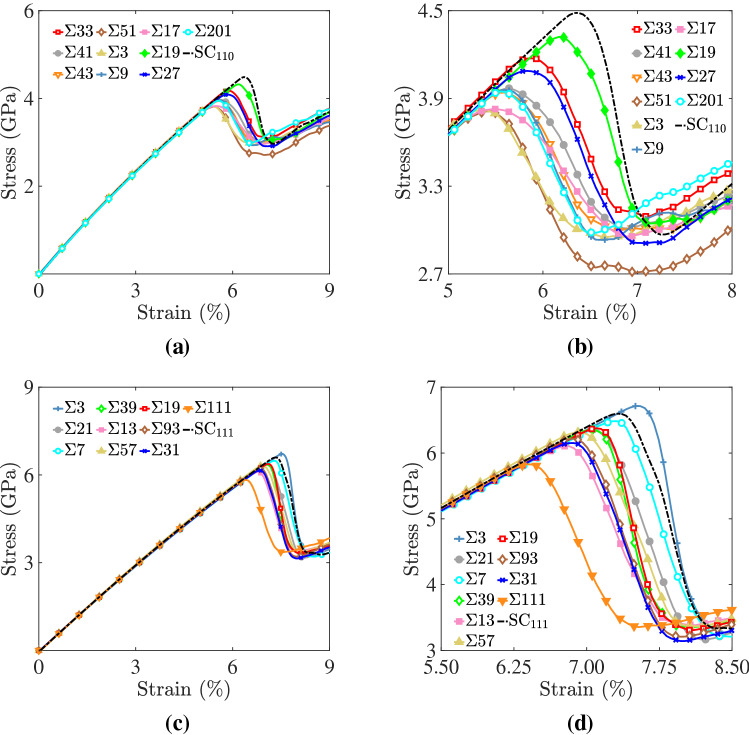
For the Cantor alloy bicrystals: **(a,c)** tensile stress–strain curves and **(b, d)** close-up views around the peak points. Curves for bicrystals with **(a,b)** [110] and (c,d) [111] STGBs are shown. Curves for Cantor alloy single crystals with [110] $(SC_{110})$ and [111] $(SC_{111})$ loading axis are shown for comparison.

### Mechanical deformation behavior

We first start by examining the macroscopic mechanical behavior of the bicrystal systems at 77 K. Figure 4 shows stress-strain curves for the Cantor alloy bicrystals with [110] (Fig. 4a, b) and [111] (Fig. 4c, d) STGBs. The behavior of Cantor alloy single crystals with [110] ($\hbox {SC}_{{110}}$) and [111] ($\hbox {SC}_{{111}}$) loading axis is included for comparison. In all systems, the tensile stress increased monotonically with strain until a peak point was reached then followed by a stress drop, the value of which was roughly 35% of the peak value. It is also noticed that the peak point in the stress–strain curve in bicrystal systems was lower than their single crystal counterparts. As will be discussed later, these stress drops are associated with partial dislocation nucleation events with GBs serving as heterogeneous nucleation sites. An exception to this is the bicrystal system with $\Sigma 3$ (111) STGB, where a close examination of Fig. 4c,d shows that the peak stress for this system is comparable to the single crystal one. This coherent $\Sigma 3$ GB is resistant to nucleation of dislocations, which in this system were found to nucleate homogeneously from the bulk crystals. For the systems with a [110] loading axis, the maximum Schmid factor for $\{111\} \langle 112 \rangle$ twinning is 0.47 and that for $\{111\} \langle 110 \rangle$ slip is 0.41, whereas for bicrystal geometries with a [111] loading axis those factors were 0.31 and 0.27 for twinning and slip, respectively. As a result, the [111] is the stiffer direction and bicrystals with a [111] loading axis attained higher stress states than those with a [110] loading axis. This effect has been recently experimentally observed in the Cantor alloy^76^. Also, Fig. 4a,c shows that in all bicrystal systems explored in this work, a plateau in the stress was observed after the initial stress drops. As will be discussed below, this plateau in the flow stress is due to the fact that further deformation was achieved by the growth of GB-nucleated defects (i.e., dislocations) into the bulk grains. Similar trends for the flow stress have been observed in several metallic systems^77,78^.

**Figure 5:**
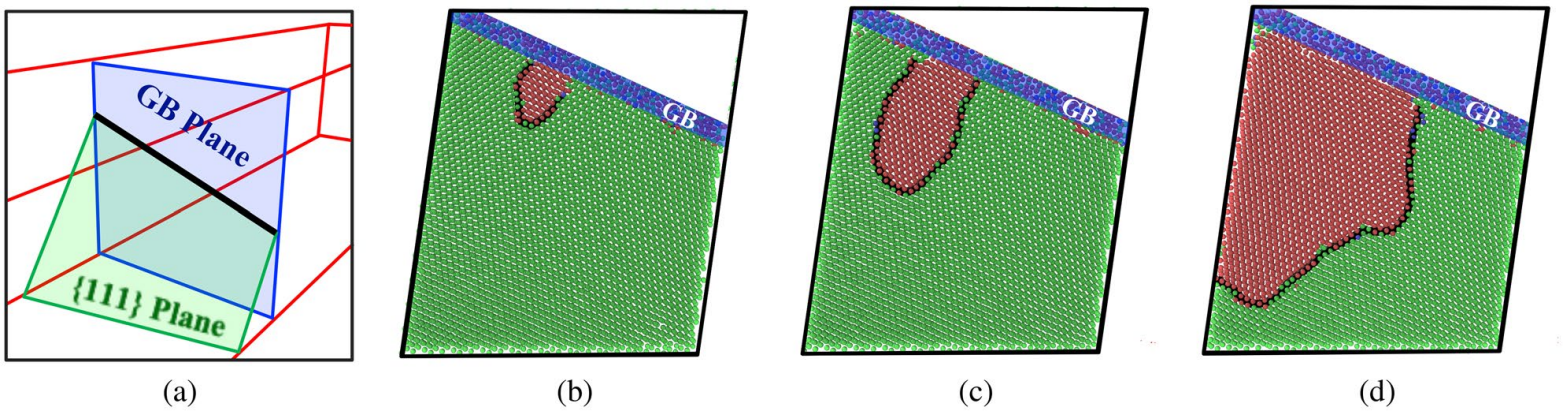
For the bicrystal with a $\Sigma 9$ [110] STGB: **(a)** a schematic representation showing the GB plane (blue) and $\{111\}$ plane (green); and **(b–d)** views along the normal to the $\{111\}$ plane depicting the nucleation and growth of a 1/6 $\langle 1 1 2 \rangle$ dislocation at a nominal strain of **(b)** $\epsilon = 5.0\%$, **(c)** $\epsilon = 5.4\%$, and **(d)** $\epsilon = 5.5\%$. In **(b)–(d)**, the black line represents the partial dislocation that demarcates regions of FCC (green) from HCP (red) atoms. The shaded region in blue denotes the intersection region of the GB and $\{111\}$ plane.

Next, we visually inspect the atomic structures of the Cantor alloy bicrystals during the course of their tensile deformation with a specific focus on tensile strains near the peak points in the stress-strain diagrams. Here, the PTM algorithm was used to identify the local structural environment around each atom and reveal GB regions and stacking faults that form during deformation. Figure 5 depicts results for the bicrystal system with a $\Sigma 9$ [110] STGB, where Fig. 5a is a schematic illustrating the intersection of a $\{111\}$ plane with this GB. Figure 5b–d shows respectively projected views normal to this $\{111\}$ plane at nominal tensile strains $\epsilon = 5.0\%, 5.4\%$ and $5.5\%$, where the GB region is labeled in blue, FCC atoms in green, and HCP ones, indicative of stacking faults, in red. A 1/6 $\langle 112 \rangle$ partial dislocation line, identified by DXA and labeled in black, was observed to nucleate from the GB and propagate into the bulk crystal leaving behind a stacking fault, and that the nucleation of this partial dislocation corresponded to the stress drop observed for this bicrystal system (refer to Fig. 4b).

**Figure 6:**
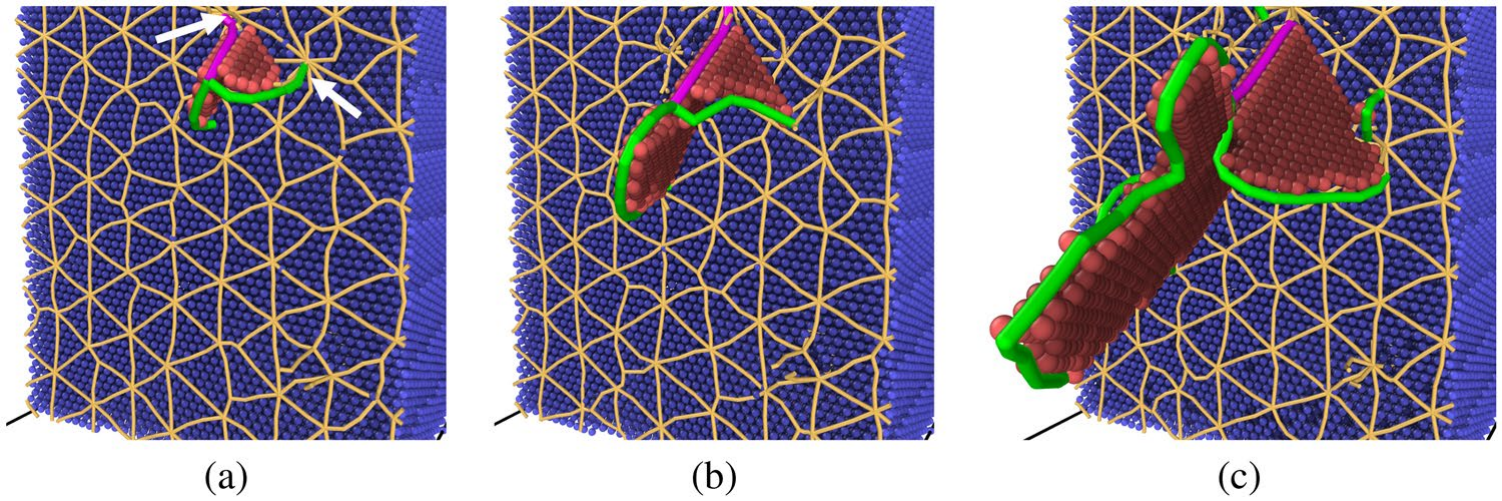
Snapshots of the bicrystal with $\Sigma 111$ [111] STGB at a nominal tensile strain of **(a)** $\epsilon = 6.1\%$, **(b)** $\epsilon = 6.2\%$, and **(c)** $\epsilon = 6.3\%$ depicting the initial stages of 1/6 $\langle 112 \rangle$ dislocation (green line) nucleation from the GB leaving behind a stacking fault (atoms in red). The orange lines represent the GB dislocation network and atoms in only one half of the bicrystal system are shown in blue for a better visualization of the defect structures.

Perspective views demonstrating the nucleation and growth of a partial dislocation in the bicrystal system with a $\Sigma 111$ [111] STGB are shown in Fig. 6. Snapshots at tensile strains $\epsilon =$ 6.1% (Fig. 6a), 6.2% (Fig. 6b) and 6.3% (Fig. 6c) are shown, where the grid of GB dislocations is colored in orange, atoms in one crystal are colored in blue, and ones in the second crystal are removed for a better visualization of the defect structures. The nucleated dislocation line is colored in green and atoms with HCP structures are in red. Supplementary Video 1 also shows an animation of this bicrystal system during the tensile deformation. At a tensile strain of $\epsilon =$ 6.1%, a 1/6 $\langle 1 1 2 \rangle$ partial dislocation line nucleated from the GB and grew by bowing out into the bulk crystal leaving behind a stacking fault. It is observed that the dislocation line has its ends pinned at two nodes of the GB dislocation network (see arrows in Fig. 6a). Again, the stress drop observed in the stress-strain curve for this bicrystal system (refer to Fig. 4d) corresponded to the nucleation of this partial dislocation from the GB. As shown in Supplementary Figs. S2 and S3 online, all bicrystal systems examined in this work exhibited similar trends to the ones depicted in Figs. 5 and 6. Such results suggest that GBs serve as heterogeneous nucleation sites for dislocations in the Cantor alloy, and upon further straining these line defects grow into the bulk crystals leaving behind faulted regions. In all bicrystal systems explored in this work, no trailing partials were observed to nucleate from the GBs. This effect was also observed in atomistic simulations of dislocation nucleation from GBs in pure metals^78,79^. Derlet and Van Swygenhoven^80^ found that the emission of first partials was enough to relieve local stresses at GBs and that the emission of trailing partials was not necessary to accommodate the applied loading.

### Deformation mechanisms

**Figure 7:**
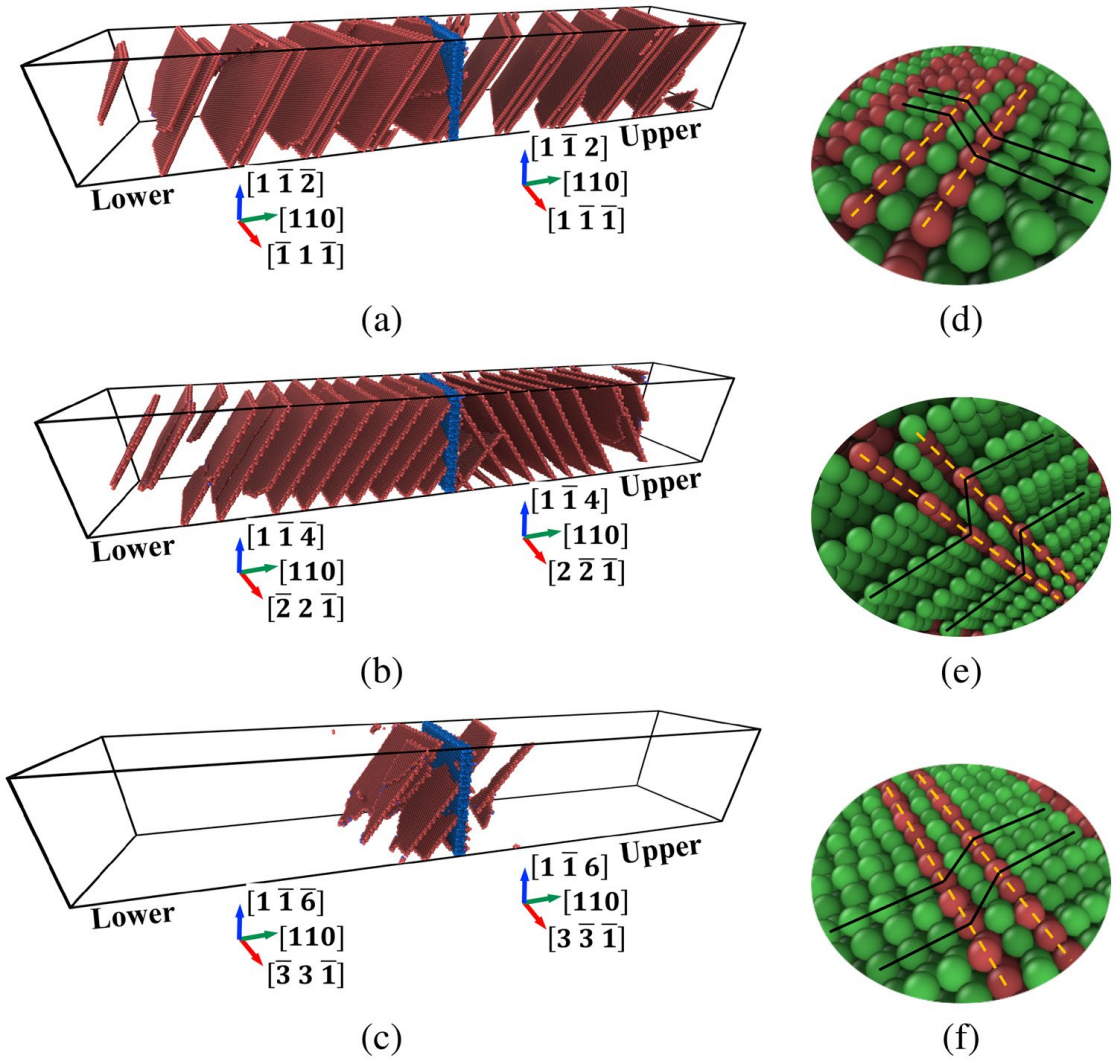
For the bicrystals with [110] STGBs: **(a)–(c)** A comparison of the deformation behavior for the systems with **(a)** $\Sigma 3$, **(b)** $\Sigma 9$, and **(c)** $\Sigma 19$ GBs at a nominal strain of $\epsilon = 6.3\%$, where red (blue) denote stacking faults (GB) atoms. Atoms with FCC ordering are removed for a better visualization of the structures. **(d)–(f)** At a nominal strain $\epsilon = 15\%$, close-up views depicting planes of faulted atoms in [110] bicrystals with **(d)** $\Sigma 3$, **(e)** $\Sigma 9$, and **(f)** $\Sigma 19$ STGBs, where atoms in green (red) denote FCC (HCP) ordering.

The mechanical behavior of the bicrystal systems is explored at late stages of deformation. Figure 7a–c shows respectively snapshots of the bicrystals with $\Sigma 3$, $\Sigma 9$, and $\Sigma 19$ [110] STGBs at a nominal tensile strain of $\epsilon = 6.3\%$. In these figures, GB and HCP atoms were labeled in blue and red, respectively, where HCP ordering is indicative of stacking faults. Atoms with FCC structures were removed to provide a better visualization of the defect structures. As can be seen from Fig. 7, the density of the nucleated partial dislocations and the resultant stacking faults at a given strain are dependent on the GBs present in these systems. For example, the bicrystal with a $\Sigma 9$ [110] STGB shown in Fig. 7b exhibits higher density of faulted regions compared to the one with a $\Sigma 19$ [110] STGB depicted in Fig. 7c. A close examination of the stress-strain curves for these bicrystals (refer to Fig. 4b) shows that the stress required to nucleate these partial dislocations was higher for the system with a $\Sigma 19$ [110] STGB than ones with $\Sigma 9$ and $\Sigma 3$ [110] GBs. At a tensile strain $\epsilon = 15\%$, Fig. 7d–f shows respectively close-up views of the atomic structures (atoms colored in green and red denote FCC and HCP structures) for the bicrystals with a $\Sigma 3, \Sigma 9$ and $\Sigma 19$ [110] STGBs, where planes of faulted atoms can be clearly seen. In all bicrystal systems examined in this work, GBs were found to facilitate the nucleation of defects (i.e., dislocations). An exception to this is the bicrystal with the coherent $\Sigma 3$ (111) STGB, where dislocations were found to nucleate homogeneously from the bulk crystals. This explains the large value of the peak stress for this system compared to the other bicrystal ones. Indeed, this behavior for the coherent $\Sigma 3$ has also been observed in other metallic systems^78^.

**Figure 8:**
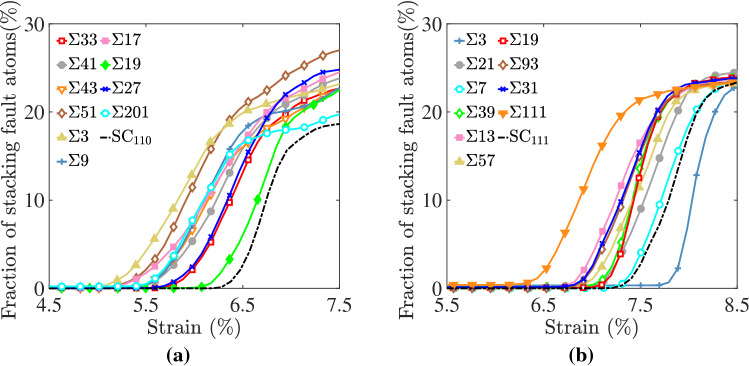
Evolution of the fraction of stacking fault atoms with respect to the total in each system as a function of tensile strain for bicrystal systems with **(a)** [110] and **(b)** [111] loading axis. Stacking fault atoms were identified using the PTM algorithm^63^.

Following the experimental approach by Laplanche et al.^42^, we quantified the microstructural evolution during mechanical deformation by calculating the fraction of stacking fault atoms with respect to the total in each system, and the results are reported in Fig. 8 for bicrystals with a [110] (Fig. 8a) and [111] (Fig. 8b) loading axis. Stacking fault atoms were identified as ones with HCP ordering using the PTM algorithm. As mentioned above, the observed stress drop in the stress–strain diagrams corresponded to the nucleation of partial dislocations from GBs, which in turn grew rapidly into the bulk crystals leaving behind faulted regions. No stacking faults were observed to nucleate prior to reaching the peak point in the stress–strain diagram. In all bicrystal systems, the rapid increase in the fraction of faulted atoms at lower strain values compared to the single crystal systems indicates that GBs serve as efficient nucleation sites for partial dislocations. The onset of rapid increase in the fraction of faulted atoms occurred at strains up to 30% lower than that for single crystal systems. Further, the fraction of atoms with local HCP structures (i.e., stacking faults) reached an asymptotic value of $\approx 25\%$ at late stages of deformation. Systems with [110] STGBs nucleated partial dislocations and experienced an increase in fraction of faulted atoms at lower strain levels compared to bicrystals with [111] STBGs. The lowest levels of applied tensile strain, leading to partial dislocation nucleation and accompanying increase in the fraction of faulted atoms occurred in bicrystals with a $\Sigma$3 [110], Fig. 8a, and $\Sigma$111 [111], Fig. 8b, STGBs.

**Figure 9:**
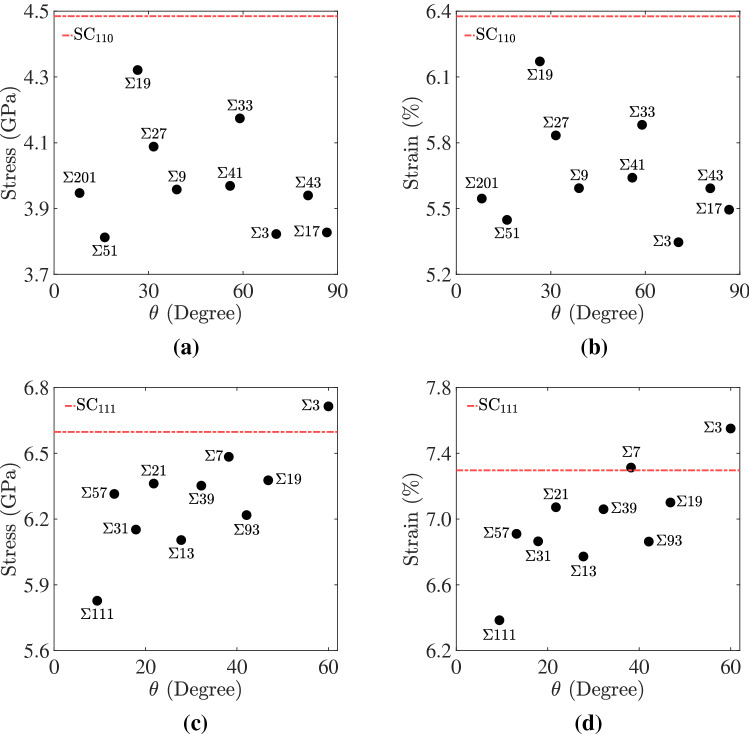
For the bicrystals with **(a,b)** [110] and **(c,d)** [111] STGBs, plots of the ultimate **(a,c)** stress and **(b,d)** strain as a function of the GBs present in these systems. Ultimate stress and strain values for Cantor alloy single crystals (dashed red lines) are also plotted for comparison.

To relate the trends observed in Figs. 4 and 8 to GB geometry, we obtained the ultimate stress and strain values from the peak point in the stress–strain diagram as a function of GB misorientation (i.e., twist angle) for all bicrystal systems, and the results are shown in Fig. 9. Again, the bicrystal ultimate stress and strain values are lower than the single crystal ones, which is an indication that GBs act as preferred sites for dislocation nucleation. A close examination of Fig. 9 also shows that bicrystals with a [111] loading axis are characterized by higher ultimate stress (cf. Fig. 9a,c) and strain (cf. Fig. 9b,d) values, i.e., more mechanical energy is required to nucleate partial dislocations from GBs in [111] bicrystals. Variations in ultimate strain and stress values also exist as a function of GB type, defined in these bicrystal systems by the twist angle $\theta$. For the bicrystals with a [111] loading axis, a trend exists, albeit with some scatter, where the ultimate stress and strain increase with the twist angle $\theta$. Further, an examination of Fig. 9c,d reveals a large difference in the nucleation stress/strain between the $\Sigma 111$ and $\Sigma 3$ GB. Owing to the small twist angle, $\theta = 9.43^{\circ }$, of $\Sigma 111$ and the well defined interfacial dislocation structure, this GB was found to be the most efficient in nucleating partial dislocations, i.e., it exhibited the smallest ultimate stress and strain values out of all [111] systems. On the other hand, dislocations were observed to nucleate homogeneously form the bulk crystals in the system with the coherent $\Sigma 3$. For the bicrystal systems with a [110] loading axis, our results suggest that ultimate stress and strain values are lower for small ($\lesssim 30^{\circ }$) and large ($\gtrsim 60^{\circ }$) twist angles compared to intermediate ones. The results depicted in Fig. 9 show that while each set of [110] and [111] bicrystal systems have the same maximum Schmid factors for slip and twinning, variations do exist due to the GBs present in these systems. The Schmid factor provides crystallographic directions with the most resolved shear stresses; however, as depicted in Fig. 9 our work shows that the presence of GBs plays a role in the dislocation nucleation process in HEAs.

## Discussion

While the Cantor alloy has been the subject of numerous studies in recent years, most of these have been mainly focused on the effects of element compositions away from the equiatomic one^26^, stacking fault energies^51,81^, and lattice distortions^82–84^. The role of GBs in the mechanical deformation of HEA alloys remains poorly understood. Tensile stress-strain diagrams for Cantor alloy bicrystals with [110] and [111] loading axes show that GBs facilitate the nucleation of partial dislocations, which with further deformation grow in the bulk crystal leaving behind stacking faults. This is in agreement with recent experimental findings demonstrating that GBs act as nucleation sites for mechanical nanotwins^38,42^. The bicrystals with a [111] loading axis were found to require more mechanical energy to nucleate dislocations from GBs compared to systems with a [110] loading axis. As shown in Figs. 5 and 6, nucleation of 1/6$\langle 112 \rangle$ dislocations in the bicrystal systems occurred at lower strain values compared to their single crystal counterparts. In addition, the local GB structure plays a role in the nucleation process. For example, the bicrystal system with a $\Sigma 111$ STGB, characterized by a grid of 1/6$\langle 112 \rangle$ screw dislocations (see Fig. 3a), was found to nucleate partial dislocations at the lowest strain level (refer to Fig. 9c,d) compared to the bicrystal systems with a [111] loading axis.

**Figure 10:**
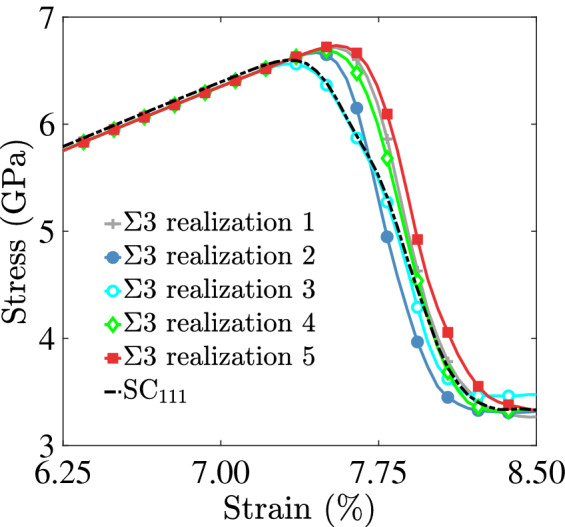
A close-up view of the stress-strain curve for several realizations of the bicrystal system with a $\Sigma 3$ [111] STGB, demonstrating the effect of local GB chemistry. The curve for the single crystal system ($\hbox {SC}_{{111}}$) is also shown.

Finally, due to the non-dilute nature of HEA compositions, it is reasonable to expect variations in the ultimate stress and strain values due to variations in the local chemistry within GB structures. To demonstrate this effect, Fig. 10 shows tensile stress-strain curves for five realizations of a Cantor alloy bicrystal with a $\Sigma 3$ [111] STGB along with that of a single crystal system with a [111] loading axis. Owing to the coherent nature of this $\Sigma 3$ GB, it was found to be the most resistant to dislocation emission. In all systems depicted in Fig. 10, dislocations nucleated homogeneously from the bulk crystals. Variations in the observed ultimate stress and strain values are due to the local GB chemistry. Our approach of generating GBs in the Cantor alloy bicrystals employed the commonly used $\gamma$-surface method^69,70^, which aims to mine for the lowest energy GB structure by performing a series of relative displacements between the upper and lower crystals in conjunction with atom deletions and energy minimizations. However, this approach does not consider chemical equilibration, which includes exploring the preferential sites within the GB for each of the elemental species, sampling the GB atomic density, and probing adsorption or desorption effects. As a result, the GB structures explored in this work can be regarded as metastable ones. Our simulation results suggest that the strength of the Cantor alloy can be manipulated by tailoring the GB types and their populations in HEA microstructures. We are currently exploring GB solute segregation in the Cantor alloy and its effect on the mechanical properties, and we hope to report on these results in a future publication.

## Supplementary information

Supplementary Information.

Supplementary Video 1.

## Data Availability

The datasets generated during and/or analyzed during the current study are available from the corresponding author on reasonable request.

## References

[1] Cantor B, Chang I, Knight P, Vincent A (2004). Microstructural development in equiatomic multicomponent alloys. Mater. Sci. Eng. A.

[2] Yeh J-W (2004). Nanostructured high-entropy alloys with multiple principal elements: novel alloy design concepts and outcomes. Adv. Eng. Mater..

[3] Schneider M (2020). Analysis of strengthening due to grain boundaries and annealing twin boundaries in the crconi medium-entropy alloy. Int. J. Plast..

[4] Ye Y, Wang Q, Lu J, Liu C, Yang Y (2016). High-entropy alloy: Challenges and prospects. Mater. Today.

[5] Zhang Y (2014). Microstructures and properties of high-entropy alloys. Prog. Mater. Sci..

[6] Zhou Y, Zhang Y, Wang Y, Chen G (2007). Solid solution alloys of Al Co Cr Fe Ni Ti X with excellent room-temperature mechanical properties. Appl. Phys. Lett..

[7] Wang X, Zhang Y, Qiao Y, Chen G (2007). Novel microstructure and properties of multicomponent cocrcufenitix alloys. Intermetallics.

[8] Tsai C-W, Tsai M-H, Yeh J-W, Yang C-C (2010). Effect of temperature on mechanical properties of al0.5cocrcufeni wrought alloy. J. Alloys Compds..

[9] Lin C-M, Tsai H-L (2011). Evolution of microstructure, hardness, and corrosion properties of high-entropy al0. 5cocrfeni alloy. Intermetallics.

[10] Chuang M-H, Tsai M-H, Wang W-R, Lin S-J, Yeh J-W (2011). Microstructure and wear behavior of alxco1. 5crfeni1. 5tiy high-entropy alloys. Acta Mater..

[11] Wu J-M (2006). Adhesive wear behavior of alxcocrcufeni high-entropy alloys as a function of aluminum content. Wear.

[12] Chen Y, Duval T, Hung U, Yeh J, Shih H (2005). Microstructure and electrochemical properties of high entropy alloys–A comparison with type-304 stainless steel. Corros. Sci..

[13] Chen S-T (2010). Microstructure and properties of age-hardenable alxcrfe1.5mnni0.5 alloys. Mater. Sci. Eng. A.

[14] Shi Y, Yang B, Liaw PK (2017). Corrosion-resistant high-entropy alloys: A review. Metals.

[15] Torbati-Sarraf H, Shabani M, Jablonski PD, Pataky GJ, Poursaee A (2019). The influence of incorporation of mn on the pitting corrosion performance of crfeconi high entropy alloy at different temperatures. Mater. Des..

[16] Wu Z, Bei H, Otto F, Pharr GM, George EP (2014). Recovery, recrystallization, grain growth and phase stability of a family of fcc-structured multi-component equiatomic solid solution alloys. Intermetallics.

[17] Gludovatz B (2014). A fracture-resistant high-entropy alloy for cryogenic applications. Science.

[18] Wu Y (2014). In-situ neutron diffraction study of deformation behavior of a multi-component high-entropy alloy. Appl. Phys. Lett..

[19] Lucas M (2012). Absence of long-range chemical ordering in equimolar fecocrni. Appl. Phys. Lett..

[20] Yao M, Pradeep KG, Tasan CC, Raabe D (2014). A novel, single phase, non-equiatomic femnnicocr high-entropy alloy with exceptional phase stability and tensile ductility. Scr. Mater..

[21] Otto F (2013). The influences of temperature and microstructure on the tensile properties of a cocrfemnni high-entropy alloy. Acta Mater..

[22] Gludovatz B (2016). Exceptional damage-tolerance of a medium-entropy alloy crconi at cryogenic temperatures. Nat. Commun..

[23] Licavoli JJ, Gao MC, Sears JS, Jablonski PD, Hawk JA (2015). Microstructure and mechanical behavior of high-entropy alloys. J. Mater. Eng. Perform..

[24] Wu S (2017). Strong grain-size effect on deformation twinning of an al0.1cocrfeni high-entropy alloy. Mater. Res. Lett..

[25] Sun S (2018). Transition of twinning behavior in cocrfemnni high entropy alloy with grain refinement. Mater. Sci. Eng. A.

[26] Choi W.-M, Jo Y. H, Sohn S. S, Lee S, Lee B.-J (2018). Understanding the physical metallurgy of the cocrfemnni high-entropy alloy: An atomistic simulation study. npj Comput. Mater..

[27] Li Z, Pradeep KG, Deng Y, Raabe D, Tasan CC (2016). Metastable high-entropy dual-phase alloys overcome the strength-ductility trade-off. Nature.

[28] Ma E, Wu X (2019). Tailoring heterogeneities in high-entropy alloys to promote strength-ductility synergy. Nat. Commun..

[29] Shabani M, Indeck J, Hazeli K, Jablonski PD, Pataky GJ (2019). Effect of strain rate on the tensile behavior of cocrfeni and cocrfemnni high-entropy alloys. J. Mater. Eng. Perform..

[30] Deng Y (2015). Design of a twinning-induced plasticity high entropy alloy. Acta Mater..

[31] Tracy CL (2017). High pressure synthesis of a hexagonal close-packed phase of the high-entropy alloy crmnfeconi. Nat. Commun..

[32] Zhang F (2017). Polymorphism in a high-entropy alloy. Nat. Commun..

[33] Laplanche G (2015). Temperature dependencies of the elastic moduli and thermal expansion coefficient of an equiatomic, single-phase cocrfemnni high-entropy alloy. J. Alloys Compds..

[34] Haglund A, Koehler M, Catoor D, George EP, Keppens V (2015). Polycrystalline elastic moduli of a high-entropy alloy at cryogenic temperatures. Intermetallics.

[35] Tian F, Varga LK, Shen J, Vitos L (2016). Calculating elastic constants in high-entropy alloys using the coherent potential approximation: Current issues and errors. Comput. Mater. Sci..

[36] Diao H, Feng R, Dahmen KA, Liaw P (2017). Fundamental deformation behavior in high-entropy alloys: An overview. Curr. Opin. Solid State Mater. Sci..

[37] Otto F, Yang Y, Bei H, George EP (2013). Relative effects of enthalpy and entropy on the phase stability of equiatomic high-entropy alloys. Acta Mater..

[38] Joo S-H (2017). Tensile deformation behavior and deformation twinning of an equimolar cocrfemnni high-entropy alloy. Mater. Sci. Eng. A.

[39] Wang B (2016). Mechanical properties and microstructure of the cocrfemnni high entropy alloy under high strain rate compression. J. Mater. Eng. Perform..

[40] He J (2014). Effects of al addition on structural evolution and tensile properties of the feconicrmn high-entropy alloy system. Acta Mater..

[41] Wu Z, Bei H, Pharr GM, George EP (2014). Temperature dependence of the mechanical properties of equiatomic solid solution alloys with face-centered cubic crystal structures. Acta Mater..

[42] Laplanche G, Kostka A, Horst O, Eggeler G, George E (2016). Microstructure evolution and critical stress for twinning in the crmnfeconi high-entropy alloy. Acta Mater..

[43] Pickering E, Jones N (2016). High-entropy alloys: A critical assessment of their founding principles and future prospects. Int. Mater. Rev..

[44] Miracle DB, Senkov ON (2017). A critical review of high entropy alloys and related concepts. Acta Mater..

[45] Gali A, George EP (2013). Tensile properties of high-and medium-entropy alloys. Intermetallics.

[46] George EP, Raabe D, Ritchie RO (2019). High-entropy alloys. Nat. Rev. Mater..

[47] Schuh B (2015). Mechanical properties, microstructure and thermal stability of a nanocrystalline cocrfemnni high-entropy alloy after severe plastic deformation. Acta Mater..

[48] Stepanov N (2015). Effect of cryo-deformation on structure and properties of cocrfenimn high-entropy alloy. Intermetallics.

[49] Smith TM (2016). Atomic-scale characterization and modeling of 60 dislocations in a high-entropy alloy. Acta Mater..

[50] Zaddach A, Niu C, Koch C, Irving D (2013). Mechanical properties and stacking fault energies of nifecrcomn high-entropy alloy. Jom.

[51] Huang S (2015). Temperature dependent stacking fault energy of fecrconimn high entropy alloy. Scr. Mater..

[52] Bell RL, Cahn RW (1957). The dynamics of twinning and the interrelation of slip and twinning in zinc crystals. Proc. R. Soc. Lond. Ser. A. Math. Phys. Sci..

[53] Bell R, Cahn R (1953). The nucleation problem in deformation twinning. Acta Metall..

[54] Han W, Wu S, Li S, Zhang ZF (2008). Origin of deformation twinning from grain boundary in copper. Appl. Phys. Lett..

[55] Wang J, Beyerlein I, Hirth J (2012). Nucleation of elementary and twinning dislocations at a twin boundary in hexagonal close-packed crystals. Model. Simul. Mater. Sci. Eng..

[56] Molnár P, Jäger A, Lejček P (2012). Twin nucleation at grain boundaries in mg-3 wt.% al-1 wt.% zn alloy processed by equal channel angular pressing. Scr. Mater..

[57] Wang L, Eisenlohr P, Yang Y, Bieler T, Crimp M (2010). Nucleation of paired twins at grain boundaries in titanium. Scr. Mater..

[58] Jin Z, Bieler TR (1995). An in-situ observation of mechanical twin nucleation and propagation in tial. Philos. Mag. A.

[59] Ding Q (2019). Real-time nanoscale observation of deformation mechanisms in crconi-based medium-to high-entropy alloys at cryogenic temperatures. Mater. Today.

[60] Plimpton S (1995). Fast parallel algorithms for short-range molecular dynamics. J. Comput. Phys..

[61] Stukowski A (2010). Visualization and analysis of atomistic simulation data with ovito-the open visualization tool. Model. Simul. Mater. Sci. Eng..

[62] Stukowski A, Bulatov VV, Arsenlis A (2012). Automated identification and indexing of dislocations in crystal interfaces. Model. Simul. Mater. Sci. Eng..

[63] Larsen PM, Schmidt S, Schiøtz J (2016). Robust structural identification via polyhedral template matching. Model. Simul. Mater. Sci. Eng..

[64] Shinoda W, Shiga M, Mikami M (2004). Rapid estimation of elastic constants by molecular dynamics simulation under constant stress. Phys. Rev. B.

[65] Randle V (1993). The measurement of grain boundary geometry.

[66] Jiang H, Szlufarska I (2018). Small-angle twist grain boundaries as sinks for point defects. Sci. Rep..

[67] Chen B-R, Yeh A-C, Yeh J-W (2016). Effect of one-step recrystallization on the grain boundary evolution of cocrfemnni high entropy alloy and its subsystems. Sci. Rep..

[68] Lin Q (2017). In-situ high-resolution transmission electron microscopy investigation of grain boundary dislocation activities in a nanocrystalline crmnfeconi high-entropy alloy. J. Alloys Compds..

[69] Mishin Y, Farkas D (1998). Atomistic simulation of [001] symmetrical tilt grain boundaries in nial. Philos. Mag. A.

[70] Suzuki A, Mishin Y (2003). Atomistic modeling of point defects and diffusion in copper grain boundaries. Interf. Sci..

[71] Frolov T, Divinski S, Asta M, Mishin Y (2013). Effect of interface phase transformations on diffusion and segregation in high-angle grain boundaries. Phys. Rev. Lett..

[72] Han J, Vitek V, Srolovitz DJ (2016). Grain-boundary metastability and its statistical properties. Acta Mater..

[73] Frolov T (2018). Grain boundary phases in bcc metals. Nanoscale.

[74] Zhu Q, Samanta A, Li B, Rudd RE, Frolov T (2018). Predicting phase behavior of grain boundaries with evolutionary search and machine learning. Nat. Commun..

[75] Wynblatt P, Chatain D (2019). Modeling grain boundary and surface segregation in multicomponent high-entropy alloys. Phys. Rev. Mater..

[76] Kireeva I, Chumlyakov YI, Pobedennaya Z, Kuksgausen I, Karaman I (2017). Orientation dependence of twinning in single crystalline cocrfemnni high-entropy alloy. Mater. Sci. Eng. A.

[77] Zhang L, Lu C, Tieu K (2014). Atomistic simulation of tensile deformation behavior of 5 tilt grain boundaries in copper bicrystal. Sci. Rep..

[78] Spearot DE, Tschopp MA, Jacob KI, McDowell DL (2007). Tensile strength of$$<$$ 1 0 0$$>$$ and$$<$$ 1 1 0$$>$$ tilt bicrystal copper interfaces. Acta Mater..

[79] Capolungo L (2007). Dislocation nucleation from bicrystal interfaces and grain boundary ledges: Relationship to nanocrystalline deformation. J. Mech. Phys. Solids.

[80] Derlet P, Van Swygenhoven H (2002). Length scale effects in the simulation of deformation properties of nanocrystalline metals. Scr. Mater..

[81] Okamoto NL (2016). Size effect, critical resolved shear stress, stacking fault energy, and solid solution strengthening in the crmnfeconi high-entropy alloy. Sci. Rep..

[82] Oh HS (2016). Lattice distortions in the feconicrmn high entropy alloy studied by theory and experiment. Entropy.

[83] Wang P, Wu Y, Liu J, Wang H (2017). Impacts of atomic scale lattice distortion on dislocation activity in high-entropy alloys. Extreme Mech. Lett..

[84] Ye Y (2018). Atomic-scale distorted lattice in chemically disordered equimolar complex alloys. Acta Mater..

