# Supplementary material for: Tensile deformation behavior of twist grain boundaries in CoCrFeMnNi high entropy alloy bicrystals
Source: Sci Rep. 2021 Jan 11;11:428. doi: 10.1038/s41598-020-77487-z (PMC7801446; doi:10.1038/s41598-020-77487-z)
Supplement: Supplementary file 1 — Supplementary Information. [file 41598_2020_77487_MOESM1_ESM.pdf]

Tensile Deformation Behavior of Twist Grain Boundaries in CoCrFeMnNi High Entropy Alloy Bicrystals

Hyunsoo Lee[1], Mitra Shabani[1], Garrett J. Pataky[1], Fadi Abdeljawad[1,2]
[1] Department of Mechanical Engineering,
Clemson University, Clemson, South Carolina, 29634, USA
[2] Department of Materials Science and Engineering,
Clemson University, Clemson, South Carolina, 29634, USA

Table S1. Dimensions of the simulation box for the bicrystal systems employed in the current work. Here, L_y is the dimension along the loading axis, and L_x and L_z are the in-plane ones.

**⟨110⟩ GBs**			
**Σ**	$\boldsymbol{L_x}$ (Å)	$\boldsymbol{L_y}$ (Å)	$\boldsymbol{L_z}$ (Å)
3	121.94	607.34	103.47
9	105.60	608.60	104.54
17	116.11	608.53	102.63
19	122.75	608.63	108.50
27	109.74	608.62	103.47
33	121.33	608.59	114.39
41	135.23	608.61	127.50
43	138.50	608.61	130.57
51	100.55	608.57	106.65
201	99.81	608.47	105.86
SC_{110}	121.33	607.32	114.39
**⟨111⟩ GBs**			
Σ	L_x (Å)	L_y (Å)	L_z (Å)
3	103.47	611.73	104.54
7	114.06	609.92	105.37
13	124.35	610.94	107.69
19	112.75	611.54	108.45
21	136.87	609.92	105.37
31	96.01	611.24	110.87
39	124.35	611.03	107.69
57	112.75	611.52	108.49
93	144.02	611.47	110.87
111	104.894	611.337	121.121
SC_{111}	103.466	609.702	119.473

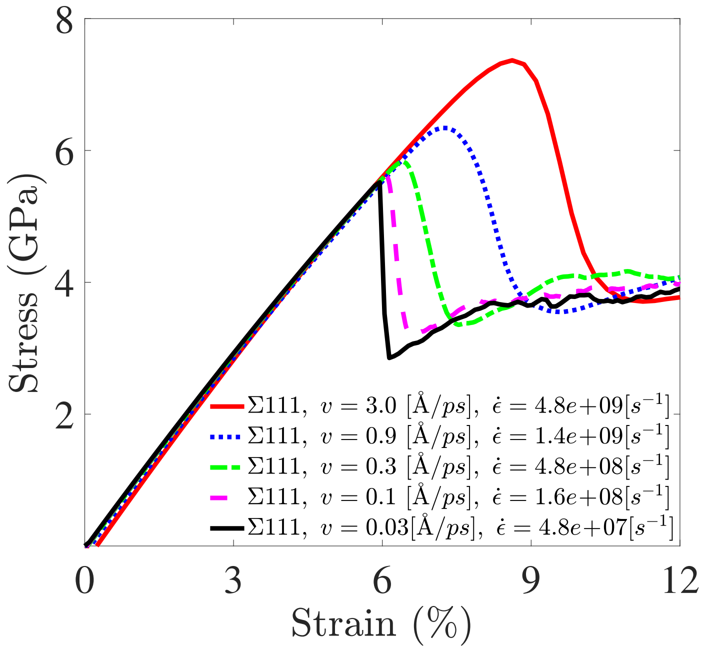

Fig. S1. For the Cantor HEA alloy bi-crystal with a Σ111 [111] symmetric twist GB, tensile stress-strain curves at different strain rates $\dot{\epsilon}$.

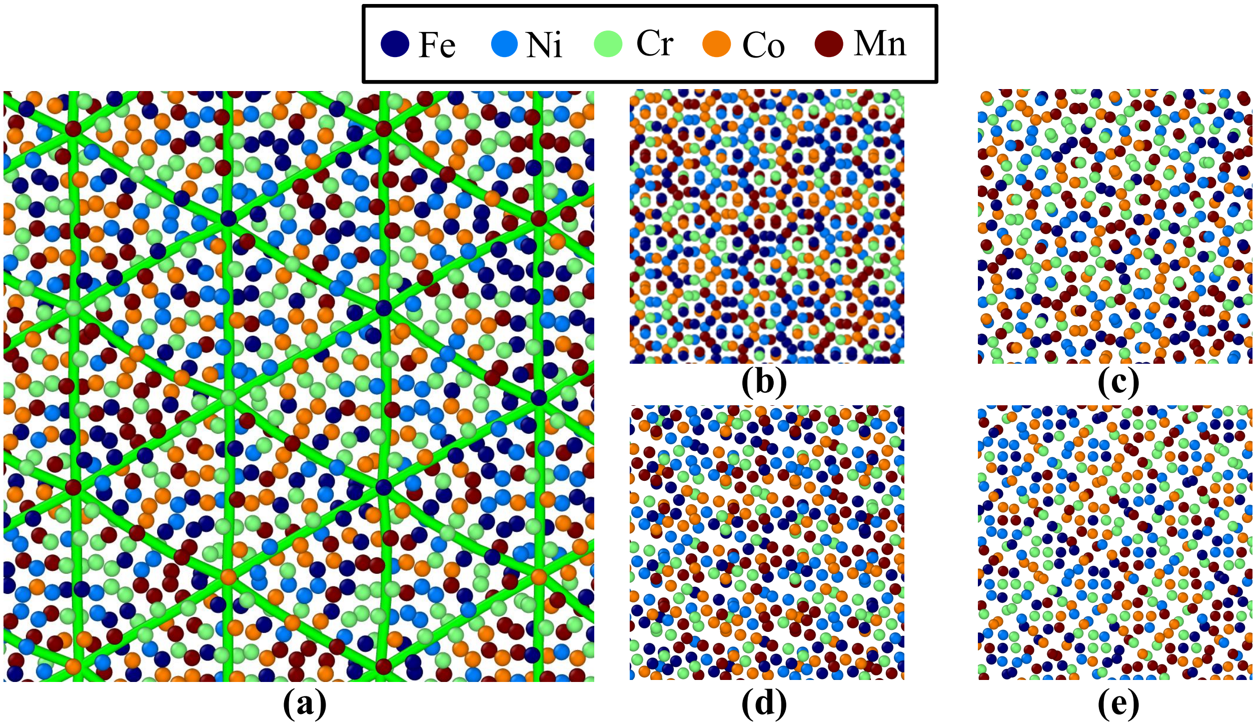

Fig. S2. View of the GB plane showing the atomic arrangement for select (a)-(c) [111] and (d)-(e) [110] symmetric twist GBs. The structure of (a) Σ111, (b) Σ7, (c) Σ39, (d) Σ9, and (e) Σ17 GBs are shown. Atoms are colored according to element type. For the Σ111 [111] STGB in (a), interfacial dislocation network (green) extracted using DXA is shown.

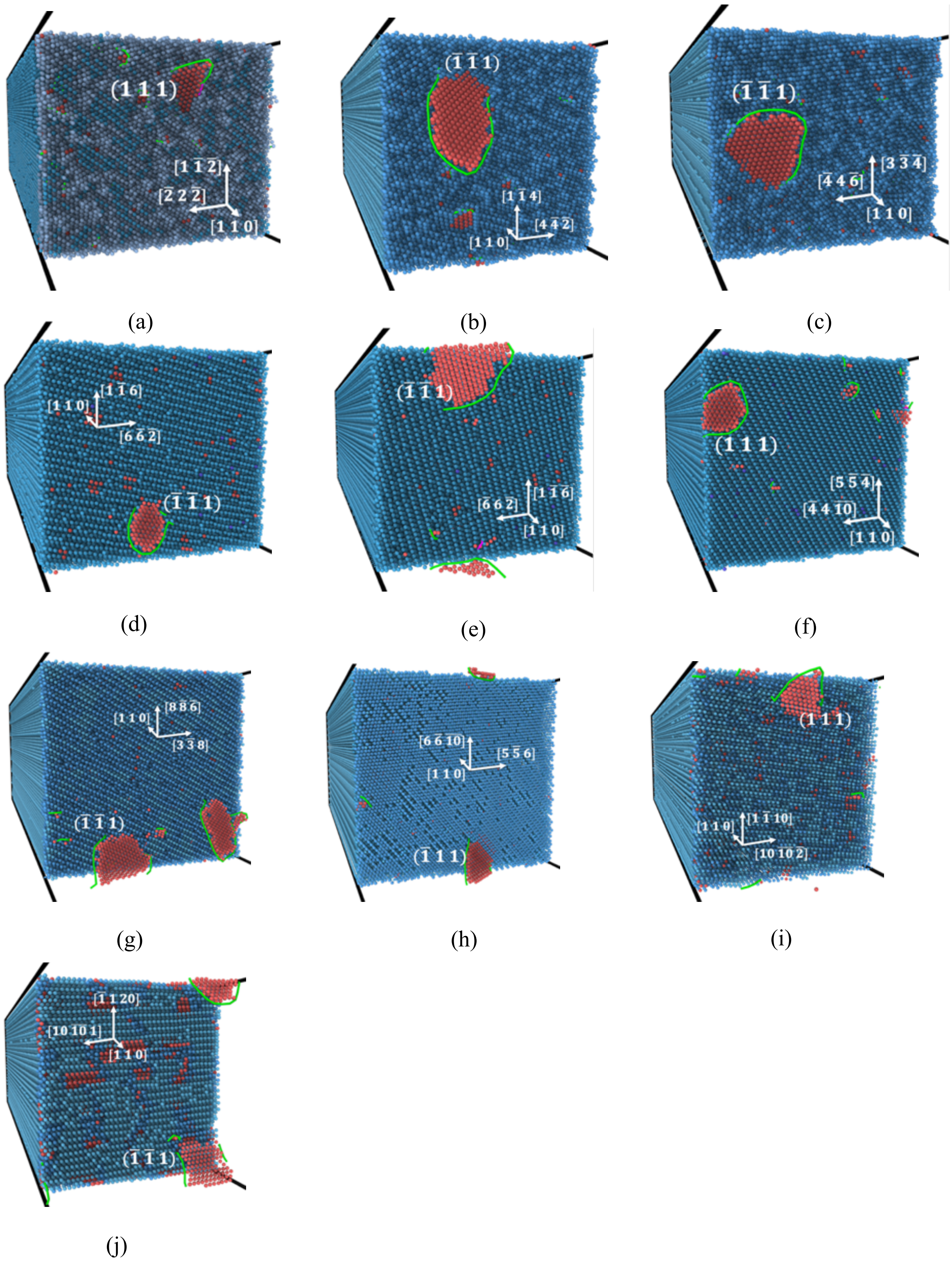

Fig. S3. For the HEA bi-crystals with (a) Σ3 (b) Σ9 (c) Σ17 (d) Σ19 (e) Σ27 (f) Σ33 (g) Σ41 (h) Σ43 (i) Σ51 and (j) Σ201 ⟨110⟩ symmetric twist GBs, snapshots depicting the onset of defect nucleation at GBs. Atoms located in one side of the bicrystal are deleted for a better visualization of the structures. Atoms colored in blue (red) denote FCC (HCP) ordering. Green lines denote partial dislocations.

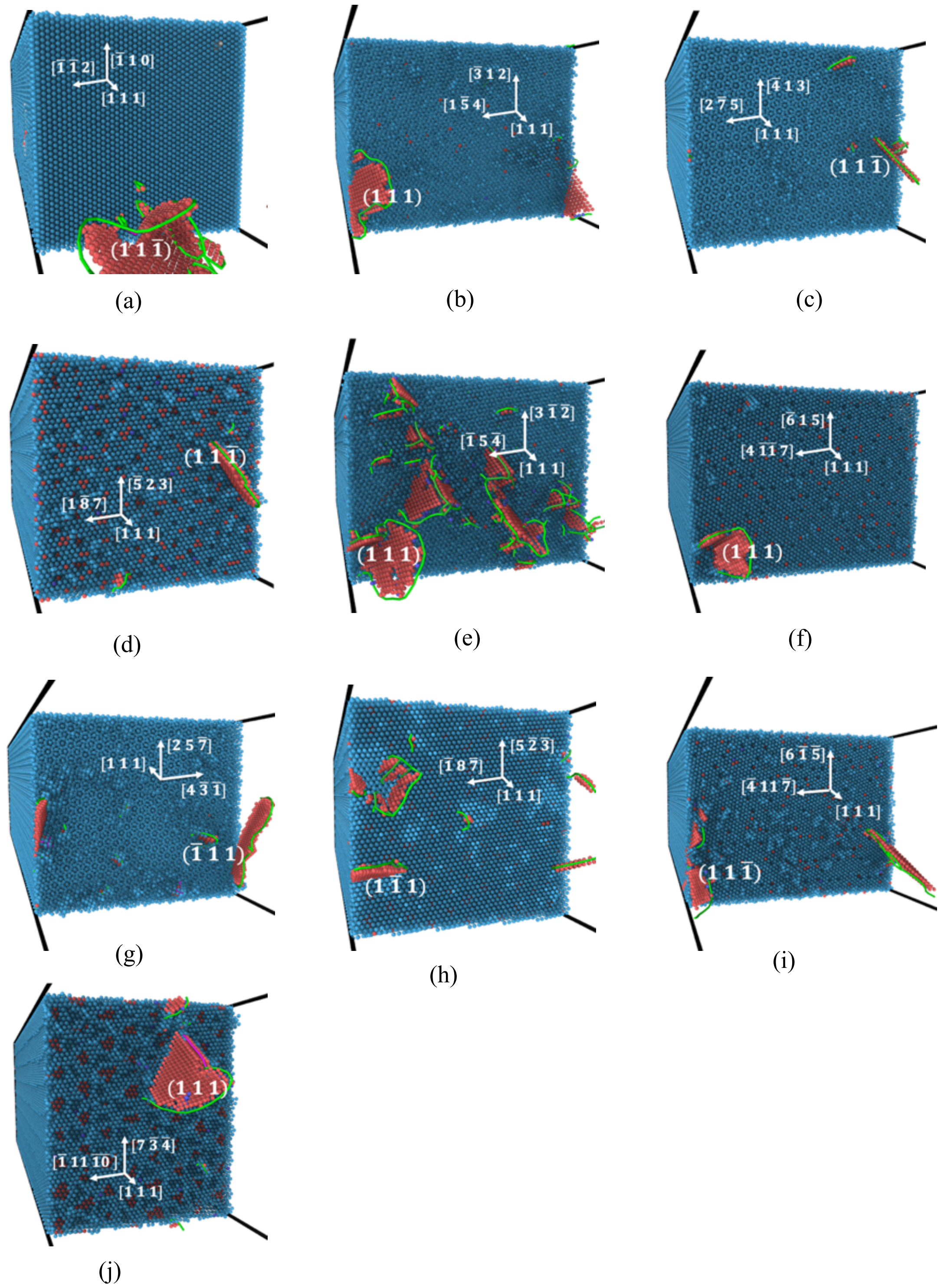

Fig. S4. For the HEA bi-crystals with (a) Σ3 (b) Σ7 (c) Σ13 (d) Σ19 (e) Σ21 (f) Σ31 (g) Σ39 (h) Σ57 (i) Σ93 and (j) Σ111 ⟨111⟩ symmetric twist GBs, snapshots depicting the onset of defect nucleation at GBs. Atoms located in one side of the bicrystal are deleted for a better visualization of the structures. Atoms colored in blue (red) denote FCC (HCP) ordering. Green lines denote partial dislocations.
